# Co-ingestion of carbohydrate and whey protein increases fasted rates of muscle protein synthesis immediately after resistance exercise in rats

**DOI:** 10.1371/journal.pone.0173809

**Published:** 2017-03-15

**Authors:** Wanyi Wang, Zhenping Ding, Geoffrey J. Solares, Soon-Mi Choi, Bo Wang, Aram Yoon, Roger P. Farrar, John L. Ivy

**Affiliations:** 1 Exercise Physiology and Metabolism Laboratory, Department of Kinesiology and Health Education, University of Texas at Austin, Austin, Texas, United States of America; 2 Department of Athletic Training and Exercise Physiology, Midwestern State University, Wichita Falls, Texas, United States of America; 3 Sports Science College, Beijing Sports University, Beijing, China; 4 Muscle Physiology Laboratory, Department of Kinesiology and Health Education, University of Texas at Austin, Austin, Texas, United States of America; West Virginia University School of Medicine, UNITED STATES

## Abstract

The objective of the study was to investigate whether co-ingestion of carbohydrate and protein as compared with protein alone augments muscle protein synthesis (MPS) during early exercise recovery. Two months old rats performed 10 repetitions of ladder climbing with 75% of body weight attached to their tails. Placebo (PLA), whey protein (WP), or whey protein plus carbohydrate (CP) was then given to rats by gavage. An additional group of sedentary rats (SED) was used as controls. Blood samples were collected immediately and at either 1 or 2 h after exercise. The flexor hallucis longus muscle was excised at 1 or 2 h post exercise for analysis of MPS and related signaling proteins. MPS was significantly increased by CP compared with PLA (p<0.05), and approached significance compared with WP at 1 h post exercise (p = 0.08). CP yielded a greater phosphorylation of mTOR compared with SED and PLA at 1 h post exercise and SED and WP at 2 h post exercise. CP also increased phosphorylation of p70S6K compared with SED at 1 and 2 h post exercise. 4E-BP1 phosphorylation was inhibited by PLA at 1 h but elevated by WP and CP at 2 h post exercise relative to SED. The phosphorylation of AMPK was elevated by exercise at 1 h post exercise, and this elevated level was sustained only in the WP group at 2 h. The phosphorylation of Akt, GSK3, and eIF2Bε were unchanged by treatments. Plasma insulin was transiently increased by CP at 1 h post exercise. In conclusion, post-exercise CP supplementation increases MPS post exercise relative to PLA and possibly WP, which may have been mediated by greater activation of the mTOR signaling pathway.

## Introduction

Resistance exercise (RE) is a potent stimulator of muscle protein synthesis (MPS), and the repeated activity can bring about skeletal muscle hypertrophy [1]. However, some previous animal studies have suggested that MPS is inhibited or unchanged early after exercise [2, 3]. When it comes to muscle protein breakdown (MPB), it was reported to be increased during RE and remain elevated for up to 24 h post exercise in the fasted state [1, 4]. Under these conditions a negative muscle net protein balance can occur during the early recovery phase following RE [4]. Therefore, nutritional supplementation immediately after exercise is crucial to increase MPS as well as inhibit MPB during the early phase of recovery. It is commonly accepted that protein supplementation provides sufficient amino acids and activation of the mammalian target of rapamycin (mTOR) signaling pathway to increase MPS after exercise [5–7]. mTOR is a central kinase that integrates upstream signals from muscle contraction, amino acid (AA), and growth factors to stimulate MPS via mediating protein translation initiation. As such, a single bout of RE is able to activate the mTOR signaling pathway, which can be further potentiated by providing a post exercise protein supplement. Interestingly, Anthony and colleagues reported that high intensity endurance exercise reduced muscle anabolism during early recovery from exercise in rats, and protein supplementation could not recover the synthetic rate back to basal level [3].

Results from studies in which carbohydrate (CHO) was added to a protein supplement implied the addition of carbohydrate could potentially benefit post exercise muscle recovery [8–10]. Immediately after high intensity RE in the fasted state, the human body is in a negative net protein balance partly due to the reduction of insulin and the elevation of catabolic hormones, including cortisol and epinephrine. Numerous studies have revealed that insulin is strongly anti-catabolic by demonstrating a suppressive effect of insulin on MPB [11–13] through inhibition of forkhead box 3A (FOXO3A) [14]. Insulin also activates the mTOR signaling pathway via activation of phosphatidylinositol 3-kinase (PI3k). Some *in vitro* studies demonstrated that insulin significantly increased myotube protein synthesis [15, 16], but only a few *in vivo* studies have found a positive effect of insulin on MPS [17, 18]. The ineffectiveness of insulin to stimulate MPS *in vivo*, may be due to a lack of AA availability as insulin increases AA clearance from the blood. However, co-ingestion of CHO and protein could possibly maximize muscle protein accretion post exercise by supplying a sufficient concentration of AA while also promoting a hyperinsulinemic state. To date, however, studies investigating the combined effects of CHO and protein/AA supplementation on MPS have reported inconsistent results [10, 19–21]. Hence, the primary objective of this study was to investigate, using a rat model, if adding CHO to a whey protein (WP) supplement results in a greater MPS during the early period of exercise recovery relative to WP alone. The present study also examined the phosphorylation state of signaling proteins in a manner that increase MPS and reduces MPB. It was hypothesized that supplementing post resistance exercise with a combination of CHO plus WP would result in a great MPS than supplementing with WP alone.

## Materials and methods

### Animals

Eighty male Sprague-Dawley rats were obtained at two months of age from Charles River (Wilmington, MA). Rats were housed two per cage and provided standard laboratory chow (Prolab RMH 1800 5LL2, LabDiet, Brentwood, MO) and water ad libitum. The temperature of the animal room was maintained at 21°C, with a reverse artificial 12:12 h dark-light cycle. Experimental procedures were approved by the Institutional Animal Care and Use Committee (IACUC) of the University of Texas at Austin and conform to the guidelines for the use of laboratory animals published by the United States Department of Health and Human Resources. We elected to use the Sprague-Dawley rat in order to tightly control diet and exercise. In addition, this rat model has a high degree of genetic homogeneity, which reduces variance in exercise response.

### Exercise familiarization

Following 1 week of acclimation, rats underwent 3 repeated sessions of ladder climbing to familiarize with the exercise protocol. Each session consisted of 4 trials and was separated by 1 day. The rats carried no weight during this familiarization period. The ladder was 1 meter height with an incline of 85° with 2 cm grid steps. After the initial familiarization, the rats then completed another 3 practice sessions of climbing separated by 1 day between each session with 50, 60 and 70% of their body mass attached to their tails, respectively. The weight was secured to the tail with foam tape (3M Conan) and a Velcro strap. Rats were encouraged to climb by lightly tapping their tails with a bottle brush.

### Experimental protocol

Following an overnight fast, rats underwent an acute resistance exercise, as described in our previous study [22]. Briefly, rats were placed on a climbing ladder to ascend 10 times with a weight equal to 75% of their body mass attached to the tail. There were two minutes rests between each climb. Upon the completion of the exercise protocol, each rat was wrapped separately in a towel. A 0.7 ml blood sample was obtained from the tips of their tails. Immediately after exercise, animals received a placebo (PLA = ddH_2_O), whey protein (WP = 0.375 g/kg), or carbohydrate plus WP (CP = 1.2 g/kg dextrose+0.375 g/kg WP) supplement by gavage. Twenty rats were used as sedentary controls (SED) and received a gavage of ddH_2_O. All rats were randomly assigned to a treatment prior exercise familiarization. Rats in each treatment group were then subdivided by time of euthanasia, which occurred at 1 or 2 h post gavage (n = 10). After the gavage, rats were returned to their respective cage. Either 30 or 90 min after gavage, 0.04 μmol/g body weight of puromycin dissolved in PBS (pH = 7.4) was given to the rats by intraperitoneal injection to determine rate of muscle protein synthesis by the surface sensing of translation (SUnSET) procedure. This procedure has been demonstrated to be a valid alternative to traditional radioisotope techniques, although the measurement of MPS by puromycin accumulation is indirect and semi-quantitative [23].

Approximately, 20 min after the puromycin injection, rats were anesthetized with an intraperitoneal injection of sodium pentobarbital (75 mg/kg of body weight). A second blood sample was collected, followed by the excision of the flexor hallucis longus (FHL) muscle at 1 or 2 h post gavage. The respiratory pattern and responsiveness of the rat during this surgical procedure were continuously monitored. If the rat made any kind of move in response to an incision, surgery was temporarily stopped and additional anesthesia provided. The FHL contains about 90% type II fibers, of which a majority are type IIx fibers [24]. The FHL was selected to analyze because it is the most responsive muscle during ladder climbing [25]. Upon removal of the FHL, the rats were euthanized by cardiac injection of sodium pentobarbital (65 mg/kg body weight). In our study, all rats were healthy and successfully completed all experimental procedures without incident including ladder climbing, gavage, puromycin injection, and surgery to remove the FHL.

### Blood analysis

All blood samples were centrifuged at 3,000 g for 10 min at 4°C with a FS-20 microtube rotor in a Sorvall RC-6 centrifuge (Thermo Fisher Scientific Inc. Waltham, MA). After centrifugation, the cleared plasma samples were stored at -80°C for later analysis of glucose, insulin, corticosterone, growth hormone (GH) and insulin-like growth factor (IGF)-1. Plasma glucose was determined using a colorimetric method, which employs glucose oxidase and a modified Trinder color reaction (no. 315, Sigma Chemical, St. Louis, MO). Plasma insulin was measured using a radioimmunoassay kit (Millipore Corporation, Billerica, MA) with CV<10%. The concentration of corticosterone was determined by an enzyme-linked immunosorbant assay kit (ELISA) with CV<10% (Enzo life sciences Inc. Ann Arbor, MI. Cat ADI-900-097). Plasma GH (Millipore Corporation, Billerica, MA) and IGF-1 (Immunodiagnostic Systems Inc., Scottsdale, AZ) were measured using ELISA kits with CV<10% and read at dual wavelengths of 450nm and 630nm.

### Western blot analysis

Immunoblot analysis was performed as previously described [22]. In brief, ~50 mg of muscle was homogenized in ice-cold homogenization buffer (20 mM HEPES, 2 mM EGTA, 50 mM sodium fluoride (NaF), 100 mM potassium chloride (KCl), 0.2 mM EDTA, 50 mM glycerophosphate, 1 mM DL-dithiothreitol (DTT), 0.1 mM phenylmethanesulphonyl fluoride (PMSF), 1 mM benzamidine, and 0.5 mM sodiumorthovandate (Na_2_VO_4_)) at a 1:8 dilution of wet weight muscle with a glass tissue grinder pestle (Corning Life Sciences, Lowell, MA; Caframo Stirrer Type RZR1, Wiarton, Ont. Canada). The homogenates were centrifuged at 14,000 g for 10 minutes at 4°C, and the supernatants were taken for measurement of total protein according to Lowry [26], and protein synthesis and the phosphorylation of designated cell signaling proteins by western blot. All supernatants were stored at -80°C until analyzed.

For western blots, supernatant samples containing 60μg protein were combined with an equal amount of Laemmli sample buffer (125mM Tris, 20% glycerol, 20% SDS, 0.25% bromophenol blue, and β-mercaptoethanol, pH 6.8) and boiled at 95°C for 15 min in order to denature the muscle proteins [27]. Then, samples were subjected to sodium dodecyl sulfate-polyacrylamide gel electrophoresis (SDS-PAGE) using 10–15% resolving gel at 130 V for 90 min or 90 V for 2.5 h (Bio-Rad Laboratories, Hercules, CA.). The resolved proteins were then electrically transferred onto a nitrocellulose membrane (pore size: 0.45 μm; GE Healthcare Life Sciences, Pittsburgh, PA.) using a wet transfer unit (Bio-Rad Laboratories, Hercules, CA.) at 90 V for 90 min. Ponceau S. (0.1% in 0.5% acetic acid) was used to verify the completeness of the transfer. The membranes were then washed in Tris-buffered saline (TBS) with 0.06% Tween20 (TTBS) to remove the Ponceau S. staining, and then the membranes were blocked in 7% nonfat milk in TTBS (blocking buffer) for 1 h at room temperature (RT). The membranes were then incubated with the appropriate primary antibody overnight at 4°C. The targeted phosphorylated proteins were mTOR (Ser2448), 70 kDa ribosomal protein S6 kinase (p70S6k) (Thr389), ribosomal protein S6 (rpS6) (Ser235/236), 4E binding protein 1 (4E-BP1) (γ isoform), protein kinase B (Akt) (Ser473), glycogen synthase kinase (GSK)-3α/β (ser21/9), eukaryotic initiation factor 2 (eIF2)-Bɛ (ser539), forkhead box (FOXO) 3A (Ser318/321), and 5' adenosine monophosphate-activated protein kinase (AMPK) (Thr172). Alpha-tubulin (α-tubulin) was used as an internal loading control. Anti-puromycin antibody was used to detect muscle protein synthesis according to Goodman et al. [23]. Antibodies for anti-p-eIF2Bɛ and anti-puromycin were purchased from EMD Millipore Corporation (EMD Millipore Corporation, Chicago, IL). All other antibodies were purchased from Cell Signaling Technology (Cell Signaling Technology, Beverly, MA). Following overnight primary antibody probing, all membranes were washed 5 min, 3 times with TTBS. The membrane incubated with anti-puromycin antibody was incubated with HRP-conjugated secondary anti-mouse IgG (EMD Millipore Corporation, Chicago, IL). All others were incubated with HRP-conjugated secondary anti-rabbit IgG (Cell Signaling Technology, Beverly, MA). After 3 additional 5 min washes, the membranes were visualized by enhanced chemi-luminescence (ECL) in accordance to the manufacture’s instructions (Perkin Elmer, Boston, MA). All membranes were stripped and re-probed for α-tubulin as an internal loading control. All western blots were performed in duplicate for each muscle sample to ensure reproducibility (CV<8%). Images were then captured using a charge-coupled device camera in a ChemiDoc system (Bio-Rad, Hercules, CA). Intensity of each band was quantified with Quantity One analysis software (Bio-Rad) and expressed as a percentage of a standard.

### Free puromycin concentration assay

Free puromycin as a precursor can be delivered to the muscle and incorporated into nascent peptide chains. The measurement of free puromycin was used to normalize the value of MPS obtained from western blot analysis in the same sample. The analysis was conducted as described previously [23] with slight modifications. Approximately, 30 mg of muscle were homogenized in ice-cold homogenization buffer at a 1:15 dilution of wet weight muscle with a glass tissue grinder pestle (Corning Life Sciences, Lowell, MA; Caframo Stirrer Type RZR1, Wiarton, Ont. Canada). A 250μl aliquot sample homogenate was precipitated with 28μl of 100% trichloroacetic acid and incubated for 30 min on ice followed by 5 min of centrifuge at 4,200g. This was followed by the addition of 15μl of Tris buffer containing 1M Tris, 3M NaCl, and 1% Tween 20 at pH 7.0 and 30μl of 5.25M NaOH to 250μl supernatant to adjust the pH to ~9.0 (8.97–9.03). Next, the samples were filtered through a >3kDa filter (Amicon Ultra-0.5ml; Millipore, Carrigtwohill, Ireland) at 14,000g for 60 min. Meanwhile, a range of standards (0–40 pmol/100μl) were made and adjusted to pH 9.0. A 100μl sample or standard was added to a 96-well amine-binding maleic anhydride activated plate (Pierce; Thermo Fisher Scientific) in duplicate and rocked overnight at 4°C. The next day, the plate was washed 4 times using PBS with 1% Tween 20 (PBST with pH7.0) and blocked with 1% BSA-PBST for 45 min at room temperature. 100μl of anti-puromycin antibody (clone 12D10, 1:38,400) was added to each well and incubate for 105 min at RT. Followed this incubation period, the wells were washed 3 times. Next, horseradish peroxidase-conjugated anti-mouse IgG Fc 2a (1:10,000; Millipore) was added to each well and incubated for 45min at RT. After another 4 washes, Ultra 3,3',5,5'-tetramethylbenzidine (TMB, Thermo Fisher) was added to each well and rocked for 15 min. The reaction was then stopped by 0.16 M sulfuric acid (Thermo Fisher). The absorbance was measured on a plate reader at a wavelength of 450nm. The concentration of free puromycin was calculated from a standard curve.

### Statistical analysis

Data obtained from western blot were analyzed as a percentage change relative to an insulin-stimulated rat tissue standard or anti-puromycin-injected rat tissue standard. A two-way analysis of variance (ANOVA) (time x treatment) was performed on a between-within mixed model design for the measurement in data obtained from plasma assays and data obtained from western blots. When a significant F test was identified, differences among means were determined using LSD post hoc analysis. Differences with p < 0.05 were considered statistically significant. All statistical analyses were performed using IBM SPSS Statistics v19.0 software (IBM Corporation, Armonk), and all data are expressed as mean ± standard error of the mean (SEM).

## Results

**Fig 1:**
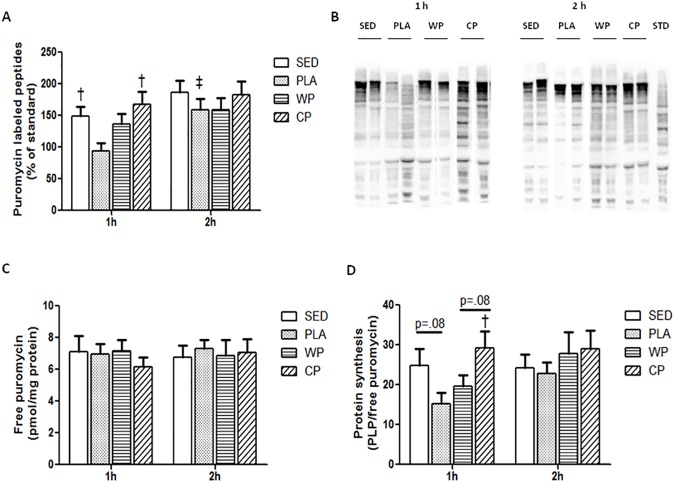
Muscle protein synthesis. (A) Quantification of puromycin-labeled peptide, expressed as a percentage of a standard sample obtained from a post exercised rat muscle. (B) Representative image of western blot analysis for puromycin using a charge-coupled device camera. For Fig 1B, each column is a western blot from a different rat randomly selected. (C) Free puromycin concentration measured as described. (D) Muscle protein synthesis expressed using the value of puromycin labeled peptides relative to the free puromycin concentration in the same sample. All values are mean ± SEM (n = 10 per group). †, p<0.05 vs. PLA. ‡, p<0.05 vs. 1 h.

In the current study, the relative value of puromycin labeled peptides from western blot analysis represented muscle protein synthesis (Fig 1A). These relative values were calculated from an image (Fig 1B) in which the intensity for puromycin signal was normalized to the anti-puromycin-injected rat tissue standard. Free puromycin is a precursor delivered to the muscle and incorporated into nascent peptide chains. There was no significant difference in levels of free puromycin among groups (Fig 1C). In order to eliminate the possibility that changes in MPS were due to the differences in precursor uptake, protein synthesis was normalized to the concentration of free puromycin in the muscle (Fig 1D). The result showed that exercise (PLA) transiently decreased MPS at 1 h post exercise compared to the sedentary (SED) (p = 0.08). However, this inhibition on MPS was completely rescued by the CP treatment (Fig 1D). CP also showed a higher trend of MPS than that of in the WP group at 1 h post exercise (p = 0.08) (Fig 1D). No significant difference was observed among treatments at 2 h post exercise (Fig 1D). (Raw data and western blot image example available in S1 File).

### Signaling pathways related MPS and MPB

**Fig 2:**
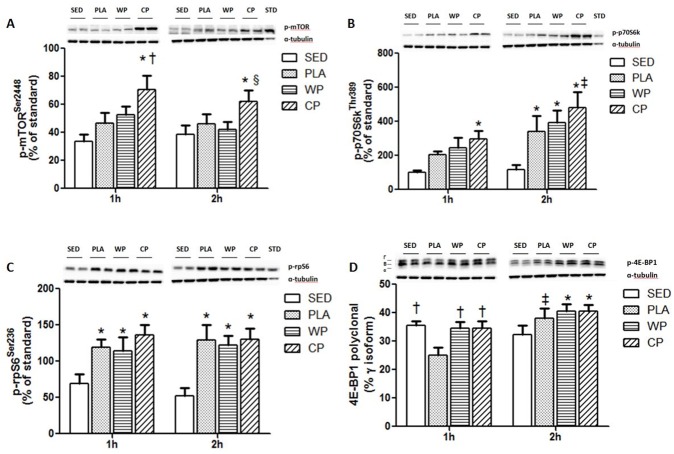
The phosphorylation of mTOR signaling pathways. (A) mTOR phosphorylation at Ser^2448^ expressed as a percentage of a standard sample obtained from an insulin-stimulated rat tissue. (B) p70S6k phosphorylation at Thr^389^expressed as a percentage of a standard sample obtained from an insulin-stimulated rat tissue. (C) rpS6 phosphorylation at Ser^236^ expressed as a percentage of a standard sample obtained from an insulin-stimulated rat tissue. (D) 4E-BP1 phosphorylation expressed as a percentage of the gamma isoform. All values are mean ± SEM (n = 10 per group).*, p<0.05 vs. SED. †, p<0.05 vs. PLA. §, p<0.05 vs. WP. ‡, p<0.05 vs. 1 h.

The phosphorylation of mTOR was significantly increased by CP at 1 and 2 h post exercise compared with SED (p<0.05). mTOR phosphorylation was also higher in CP than PLA at 1 h, and higher than WP at 2 h (Fig 2A). p70S6k phosphorylation was significantly elevated by CP at 1 h post exercise compared with SED. At 2 h, p70S6k phosphorylation was further elevated by CP, but it did not differ from that in the PLA or WP (Fig 2B). rpS6 is a downstream factor of p70S6k. Exercise significantly enhanced the phosphorylation of rpS6 both at 1 and 2 h post exercise (p<0.05). There was no difference in levels of rpS6 phosphorylation among all three exercise groups (Fig 2C). The phosphorylation of 4E-BP1 was reduced by PLA at 1 h post exercise (p<0.05). WP and CP reversed this reduction completely at 1 h and further increased the phosphorylation of 4E-BP1 at 2 h compared with SED (p<0.05). The phosphorylation of 4E-BP1 also returned to the basal level at 2 h in the PLA group (Fig 2D). (Raw data and western blot image examples available in S1 File).

**Fig 3:**
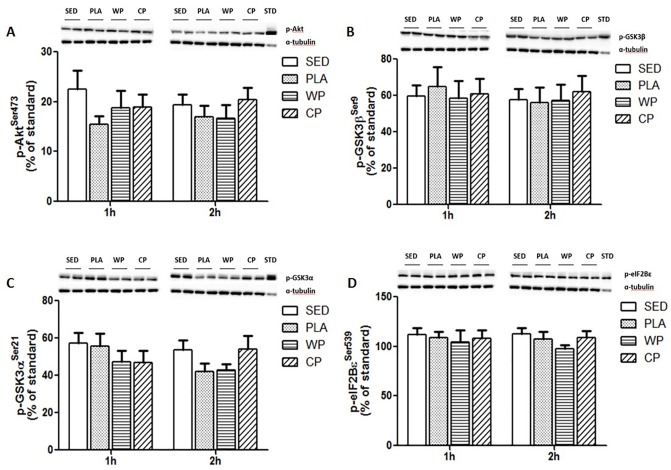
The phosphorylation of Akt-GSK signaling pathways. (A) Akt phosphorylation at Ser^473^ expressed as a percentage of a standard sample obtained from insulin-stimulated rat tissue. (B) GSK3β phosphorylation at Ser^9^ expressed as a percentage of a standard sample obtained from insulin-stimulated rat tissue. (C) GSK3α phosphorylation at Ser^21^expressed as a percentage of a standard sample obtained from insulin-stimulated rat tissue. (D) eIF2Bε phosphorylation at Ser^539^expressed as a percentage of a standard sample obtained from insulin-stimulated rat tissue. All values are mean ± SEM (n = 10 per group).

Akt is an upstream substrate of mTOR, but the phosphorylation of Akt did not show the same pattern of phosphorylation as mTOR. Akt phosphorylation did not differ statistically across treatments at 1 or 2 h post exercise (p>0.05) (Fig 3A). GSK3 is a downstream substrate of Akt. Neither GSK3α nor β showed any significant differences among groups (Fig 3B and 3C). Similarly, no significant difference in eIF2Bε phosphorylation was observed among groups at either 1 or 2 h post exercise (Fig 3D). (Raw data and western blot image examples available in S1 File).

**Fig 4:**
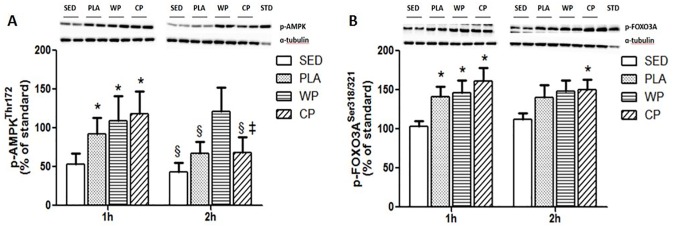
The phosphorylation of AMPK-FOXO3A signaling pathways. (A) AMPK phosphorylation at Thr^172^ expressed as a percentage of a standard sample obtained from an insulin-stimulated rat tissue. (B) FOXO3A phosphorylation at Ser^318/321^ expressed as a percentage of a standard sample obtained from an insulin-stimulated rat tissue. All values are mean ± SEM (n = 10 per group).*, p<0.05 vs. SED. §, p<0.05 vs. WP. ‡, p<0.05 vs. 1 h.

AMPKα is an energy-sensing protein. During and immediately after RE, the activation of AMPKα can reduce MPS by inhibiting mTOR, and increase MPB partly by the stimulation of FOXO3A. In the current study, the phosphorylation of AMPKα and FOXO3A was significantly increased in all three exercise groups at 1 h post exercise (Fig 4A and 4B). The phosphorylation of AMPK remained elevated at 2 h in WP (p<0.05) (Fig 4A). The phosphorylation of FOXO3A was significantly increased in the CP compared with that in SED at 2 h post exercise (p<0.05) (Fig 4B). (Raw data and western blot image examples available in S1 File).

### Glucose level and hormonal changes in plasma

There was no significant difference in plasma glucose among groups immediately post exercise (p>0.05) (Table 1). At 1 h post exercise plasma glucose was higher in the CP than that in the WP group. At 2 h post exercise plasma glucose was significantly reduced in both PLA and WP compared with immediately after exercise, and these levels were lower than that in CP (p<0.05) (Table 1). There was no difference in plasma insulin levels among groups immediately post exercise (p>0.05). However, plasma insulin was transiently elevated in the CP group at 1 h post exercise (p<0.05) (Table 1). Plasma GH was significantly reduced immediately post exercise, but it was not different among groups at either 1 or 2 h post exercise (Table 1). Plasma IGF-1 did not differ across treatments at any time point (Table 1). Plasma corticosterone was significantly elevated by exercise (p<0.05), but returned to the basal level at 1 and 2 h post exercise (Table 1). (Raw data available in S2 File).

**Table 1:** Plasma glucose, insulin, GH, IGF-1, and corticosterone concentration at 0, 1, and 2 h post exercise.

	Treatment			
	SED	PLA	WP	CP
Glucose (mM)				
0 h	5.37±0.19	5.78±0.19	5.73±0.20	5.88±0.22
1 h	5.67±0.13	5.63±0.27	5.11±0.22	6.05±0.25 §
2 h	5.11±0.27	4.71±0.27ƒ	4.22±0.25* ƒ ‡	5.69±0.29 †§
Insulin (pM)				
0 h	218.45±19.01	199.53±11.97	274.84±23.47	265.59±53.29
1 h	289.47±27.40	225.99±30.86	244.79±32.30	423.01±55.34†§ ƒ
2 h	266.43±33.46	255.51±39.97	280.76±26.16	320.64±38.30
GH (ng/ml)				
0 h	9.61±2.90	0.80±0.22*	1.46±0.53*	1.83±0.55*
1 h	6.54±2.65	3.83±1.21	9.44±7.68	9.77±5.67
2 h	3.89±0.73	7.50±2.54	7.13±3.88	2.24±0.85
IGF-1 (ng/ml)				
0 h	815.89±30.03	843.49±45.87	760.91±23.71	809.29±46.81
1 h	823.43±53.42	712.86±50.44	677.50±38.37	696.20±55.40
2 h	723.41±55.82	772.45±47.33	729.08±46.57	700.11±58.99
Corticosterone (ng/ml)				
0 h	144.84±14.63	270.94±16.84*	240.67±19.83*	227.61±25.77*
1 h	157.33±14.90	169.57±13.08 ƒ	161.99±27.01 ƒ	155.65±26.06 ƒ
2 h	117.35±17.47	127.43±14.78 ƒ	116.13±21.80 ƒ	119.12±19.49 ƒ

Data are presented as mean ± SEM (n = 10 per group).

ƒ, p<0.05 vs. 0 h in the same treatment.

‡, p<0.05 vs. 1 h in the same treatment.

*, p<0.05 vs. SED at the same time point.

†, p<0.05 vs. PLA at the same time point.

§, p<0.05 vs. WP at the same time point.

## Discussion

It is commonly accepted that RE can induce muscle protein accretion primarily by stimulating MPS, and that this activation can remain elevated for many hours after a single bout of RE [1, 17, 28]. However, within a short period of exercise recovery, MPS may be dampened [2, 3] and the net protein balance remains negative in the fasted state [1–3]. This is likely due to a lack of substrate availability. Therefore, when the interval between exercise sessions is very short, an effective muscle recovery process may be of significant benefit. The major finding of this study is that co-ingestion of CHO plus WP rescued the reduction of MPS by exercise during the early recovery phase, and was associated with the activation of the mTOR signaling pathway in the FHL of the rat.

At 1 h post exercise, MPS decreased when no supplement was provided. This finding is in agreement with that of Anthony et al. [3] using a rat endurance exercise model, but does not agree with several human studies in which resistance exercise increased muscle protein synthesis during the first hour of recovery [1, 29, 30]. It is possible that rats do not respond to resistance exercise in the same manner as humans. Another possibility for this difference in response may lie with the exercise protocol used. In the current study rats were ladder climbing to near exhaustion using muscles in their upper and lower body. However, in human studies in which MPS was increased during the first hour post exercise, the exercise was restricted to the upper leg muscles [29,30]. Recently, Macnaughton et al. found that to maximize MPS after a whole body resistance exercise protocol required a larger protein supplement than previously found when resistance exercise was performed with a relatively small muscle mass [31]. This suggests that when a large muscle mass under goes intense exercise, post exercise amino acid uptake is spread across a greater muscle mass requiring a larger protein supplement to reach maximal effectiveness. It is also possible that a whole-body exhaustive exercise such as ladder climbing could have additional consequences such as depleting substrate availability thereby delaying muscle protein synthesis until sufficient substrate is made available. We propose that the rigorous whole-body protocol used in are study may have depleted substrate availability and increased exogenous substrate dependency, and thereby dampened post exercise MPS rather than facilitating it. However, further studies are needed to identify the different acute responses to resistance exercise between rodents and humans.

The decrease in MPS observed was likely mediated through the elevated activation of AMPK and inactivation of 4E-BP1. Researchers have suggested that AMPK activation might contribute to the inhibition of MPS during exercise and to the delayed activation of MPS during early post exercise recovery [29, 32]. In the present study, phosphorylation of AMPK and inhibition of 4E-BP1 were significantly increased in PLA at 1 h post exercise relative to the SED group. However, the differences between these two groups disappeared at 2 h post exercise. The changes in these signaling proteins displayed a pattern similar to the changes in MPS when comparing the SED with PLA groups.

When supplementation was provided immediately after exercise, WP did not completely recover MPS at 1 h post exercise. However, it is worth noting that CP supplementation significantly augmented MPS compared with that of PLA at 1 h post exercise. Moreover, MPS following CP showed a tendency to be higher than either the SED and WP groups at 1 h post exercise. In a previous human research study, in which MPS was determined indirectly, the independent and combined effects of AA and CHO were compared over the initial 3 h post exercise with the finding that the combined effect of AA and CHO on MPS was roughly equal to the sum of their independent effects [10]. Although not direct proof of muscle protein synthesis, studies from our laboratory have found that post exercise CP supplementation, compared with CHO or protein intake individually, had a greater effect on the activation of anabolic signaling proteins [8, 9]. At 2 h post exercise, we did not observe any difference in MPS between the CP and other treatment groups. Perhaps, the amount of CP provided was not optimal, or the effect of CP supplement on MPS in the rat is very rapid and occurs only during the first hour of recovery unless additional supplementation is supplied at a later period.

It should be noted, that some investigators were unable to find any beneficial effect of adding CHO to a post exercise protein supplementation on MPS. For example, Koopman and colleagues compared casein plus different doses of CHO (0, 0.15, 0.6 g/kg) on whole body protein synthesis and breakdown 6 h after RE [19]. They did not observe any differences in either protein synthesis or breakdown among treatments. Similarly, Staples et al. reported neither MPS nor MPB could be further influenced by the addition of CHO to a WP supplement during the first 3 h of recovery from a single leg RE protocol [20]. The discrepancy among studies might be partly due to the types of protein intake, timing of protein synthesis measurement, dosages of supplementation, and animal model selection. Nevertheless, our results suggest that it might be prudent to reassess the effects of adding carbohydrate to a post exercise protein supplement on MPS in humans under a more comprehensive resistance exercise protocol.

With regards to the underlying mechanisms for the changes in MPS, mTOR is considered an essential kinase in the regulation of MPS. Once mTOR is activated, it further phosphorylates two downstream factors, p70S6k and 4E-BP1 [33]. p70S6k can further activate rpS6, which then increases capacity of protein synthesis. The present study showed that CP provided immediately post exercise yielded a greater increase in the phosphorylation of mTOR and p70S6k relative to the SED group at 1 h. The phosphorylation of mTOR was also higher in the CP compared with PLA at 1 h and approached being significantly higher than WP. At 2 h post exercise, phosphorylation of mTOR for CP was significantly greater than SED and WP. Phosphorylation of p70S6K in CP was significantly increased above SED at 1 and 2 h post exercise. Previous research has indicated that an EAA+CHO supplement stimulated a greater MPS post exercise than placebo and this MPS was related to a greater phosphorylation of mTOR and p70S6k [9, 30]. Based on our findings and previous results, we speculate that the increased MPS stimulated by CP was mediated by the activation of the mTOR signaling pathway. However, the actual influence the mTOR pathway had on MPS cannot be determined under the present experimental design.

We also found that exercise alone led to phosphorylation of rpS6 at both 1 and 2 h post exercise, which was not further affected by nutritional supplementation. This result was not surprising because other studies have provided strong evidence that muscle contraction is able to activate rpS6 directly via 90-kDa ribosomal S6 kinase (p90^RSK^) and without affecting p70S6k [22, 34–36]. Conversely, phosphorylation of 4E-BP1 results in its deactivation and facilitates binding of mRNA to the 40S ribosomal subunit [33]. As mentioned previously, phosphorylation of 4E-BP1 at 1 h post exercise was significantly restrained when no supplement was provided, but phosphorylation returned to the basal level at 2 h post exercise. When the supplements were provided, however, both WP and CP triggered a higher phosphorylation of 4E-BP1 relative to PLA at 1 h post exercise, and at 2 h post exercise phosphorylation levels were even higher than SED. The results suggest that WP and CP supplementations were capable of deactivating 4E-BP1 during the first hour of recovery and to dampen its activity for several hours.

Akt is an upstream substrate of mTOR. Akt can be phosphorylated via the insulin-dependent signaling pathway by phosphoinositide 3 (PI3)-kinase. The present study found that CP transiently increased plasma insulin levels at 1 h, whereas phosphorylation of Akt was not affected by any treatment. This suggests that the activation of mTOR by CP was not regulated through the insulin-signaling pathway. However, the phosphorylation and activation of Akt can be rapid and very transit. Therefore, there is the possibility that by 1 h post exercise it was too late to observe the phosphorylation of this protein. Glycogen synthase kinase (GSK) 3 is involved in another step of translation initiation. Both phosphorylated Akt and p70S6k are capable of inhibiting GSK3α/β, leading to activation of eIF2Bɛ [37, 38]. We, however, did not observe any changes in the phosphorylation levels of GSK3β, GSK3α, or eIF2Bɛ across the various treatments or times. These results are in agreement with previous findings [35, 39]. In accordance with previous research [39–41], it is possible that the regulation of MPS by the GSK3-eIF2Bɛ dependent signaling pathway only occurs during the later stages of exercise recovery. Therefore, our results indicate that during the early phase of recovery, the activation of mTOR and inhibition of 4E-BP1, regulated by nutritional supplementation, play a more important role in the stimulation of MPS than regulation of the GSK3-eIF2Bɛ signaling pathway.

AMPK is another upstream regulator of mTOR. In the present study, the phosphorylation of AMPK was significantly elevated at 1 h post exercise. This observation is supported by previous studies demonstrating that exercise alone can increase the phosphorylation of AMPK, and reduce MPS via inhibiting mTOR and decreasing 4E-BP1 phosphorylation [29, 42]. Thus, the increased AMPK phosphorylation at 1 h post exercise may count for the lack of change in the mTOR phosphorylation and the dampened MPS in the PLA and WP groups as compared to SED. In contrast, the mTOR phosphorylation and MPS for CP were significantly increased at 1 h post exercise although AMPK phosphorylation was also increased. It is speculated that the elevation in plasma insulin following the CP supplement overrode the inhibitor effects of AMPK on mTOR via the insulin-signaling pathway. It is also possible that an increase in plasma insulin can increase muscle blood flow and muscle AA transport, and block muscle AA output [43, 44]. Hence, the early enhancement of MPS by CP could have resulted from a rapid increase of AA uptake, particularly L-leucine uptake, which in turn could have activated mTOR. Further research is required to confirm either hypothesis.

Other growth factors were also measured in our study. Plasma GH was dampened immediately post exercise, but no differences were observed at 1 and 2 h post exercise among all treatment groups. It was also observed that either exercise nor nutrient supplementation affected IGF-1 plasma levels. Therefore, CP induced MPS was unlikely controlled by these hormones.

With regards to MPB, FOXO3A is a key transcription factor regulating the expressions of two muscle specific E3 ligases, muscle atrophy F-box (MAFbx or atrogin1) and muscle ring-finger protein 1 (MuRF1). These ligases have been shown to enhance muscle proteolysis [45]. In the present study, the phosphorylation of FOXO3A was significantly elevated in all three exercise groups at 1 h post exercise, but only the CP group showed sustained phosphorylation of FOXO3A compared to the SED group at 2 h post exercise. Akt and AMPK mediate the phosphorylation of FOXO3A, but with opposing control over MPB [46, 47]. In our results, Akt remained unchanged across time and treatment, and phosphorylation of AMPK did not follow the same pattern as FOXO3A. Based on these results, supplementation did not appear to inhibit MPB during the early phase of exercise recovery.

In summary, adding CHO to a protein supplement accelerates MPS during the early period of exercise recovery compared with PLA and possibly WP in the rat. This early enhancement in MPS could have been caused by a rise in plasma insulin, which in turn could have overridden the inhibitory effect of AMPK on the mTOR signaling pathway. Additional time course studies are warranted to determine changes in both MPS and MPB during extended recovery times. A limitation of our study is that we were only able to detect relative total protein changes using the puromycin procedure to assess changes in MPS. Whether or not the synthesis in contractile proteins, particular myofibrillar proteins, is elevated by CP remains to be determined. Although the exact mechanism is not known, our findings suggest that CP may be more efficacious immediately post resistance-exercise than protein alone. Caution should be taken, however, when interpreting our results due to possible differences in response to exercise and supplementation between rats and humans.

## Supporting information

S1 File Dataset and western blot image examples for MPS and the signaling proteins. (XLSX)

S2 File Dataset for blood glucose, insulin, GH, IGF-1, and corticosterone. (XLSX)
